# Three *Scrophularia* Species (*Scrophularia buergeriana*, *S. koraiensis*, and *S. takesimensis*) Inhibit RANKL-Induced Osteoclast Differentiation in Bone Marrow-Derived Macrophages

**DOI:** 10.3390/plants9121656

**Published:** 2020-11-26

**Authors:** Hyeon-Hwa Nam, A Yeong Lee, Yun-Soo Seo, Inkyu Park, Sungyu Yang, Jin Mi Chun, Byeong Cheol Moon, Jun-Ho Song, Joong-Sun Kim

**Affiliations:** Herbal Medicine Resources Research Center, Korea Institute of Oriental Medicine, 111, Geonjae-ro, Naju-si 58245, Korea; hhnam@kiom.re.kr (H.-H.N.); lay7709@kiom.re.kr (A.Y.L.); sys0109@kiom.re.kr (Y.-S.S.); pik6885@kiom.re.kr (I.P.); sgyang81@kiom.re.kr (S.Y.); jmchun@kiom.re.kr (J.M.C.); bcmoon@kiom.re.kr (B.C.M.)

**Keywords:** *Scrophularia buergeriana*, *S. koraiensis*, *S. takesimensis*, harpagoside, osteoclast differentiation, RANKL

## Abstract

Scrophulariae Radix, derived from the dried roots of *Scrophularia ningpoensis* Hemsl. or *S. buergeriana* Miq, is a traditional herbal medicine used in Asia to treat rheumatism, arthritis, and pharyngalgia. However, the effects of *Scrophularia buergeriana*, *S. koraeinsis*, and *S. takesimensis* on osteoclast formation and bone resorption remain unclear. In this study, we investigated the morphological characteristics and harpagoside content of *S. buergeriana*, *S. koraiensis*, and *S. takesimensis*, and compared the effects of ethanol extracts of these species using nuclear factor (NF)-κB ligand (RANKL)-mediated osteoclast differentiation. The harpagoside content of the three *Scrophularia* species was analyzed by high-performance liquid chromatography–mass spectrometry (HPLC/MS). Their therapeutic effects were evaluated by tartrate-resistant acid phosphatase (TRAP)-positive cell formation and bone resorption in bone marrow-derived macrophages (BMMs) harvested from ICR mice. We confirmed the presence of harpagoside in the *Scrophularia* species. The harpagoside content of *S. buergeriana*, *S. koraiensis*, and *S. takesimensis* was 1.94 ± 0.24 mg/g, 6.47 ± 0.02 mg/g, and 5.50 ± 0.02 mg/g, respectively. Treatment of BMMs with extracts of the three *Scrophularia* species inhibited TRAP-positive cell formation in a dose-dependent manner. The area of hydroxyapatite-absorbed osteoclasts was markedly decreased after treatment with the three *Scrophularia* species extracts. Our results indicated that the three species of the genus *Scrophularia* might exert preventive effects on bone disorders by inhibiting osteoclast differentiation and bone resorption, suggesting that these species may have medicinal and functional value.

## 1. Introduction

An imbalance between osteoclasts and osteoblasts affects bone formation, leading to weakened bone and the development of skeletal diseases such as osteoporosis, rheumatoid arthritis, lytic bone metastases, and chronic obstructive pulmonary disease. The function of osteoclasts, multinucleated giant cells, is bone resorption, but osteoporosis can occur if bone resorption exceeds formation due to an increase in the number of osteoclasts [1]. Most drugs used to treat osteoporosis inhibit osteoclast differentiation to control bone resorption. Receptor activator of nuclear factor (NF)-κB ligand (RANKL), a major osteoclastogenic molecule, is a member of the tumor necrosis factor (TNF) superfamily and is the initial stimulator of osteoclast differentiation, inducing the expression of osteoclast-associated genes, such as tartrate-resistant acid phosphatase (TRAP) [2,3]. Therefore, bisphosphonates and anti-RANKL antibodies that inhibit osteoclast activity are currently used for the treatment of bone resorption diseases.

Scrophulariae Radix, an herbal medicine known as Korean Hyun-Sam, is derived from the dried roots of *Scrophularia ningpoensis* Hemsl. or *S. buergeriana* Miq., plants which are widely distributed throughout the temperate regions of the Northern Hemisphere, including Asia, Europe, and North America [4,5,6]. Scrophulariae Radix has been traditionally used as a therapeutic agent for blood cooling, yin nourishing, fire pursing, and toxin removal, and is widely used to treat rheumatism and arthritis in Southwest Asia [7,8,9,10]. It has also been reported to have neuroprotective, anti-inflammatory, anti-allergy, anti-amnesia, antioxidant, and hepatoprotective effects [11,12,13,14,15]. In Korea, *S. koraiensis* Nakai (Korean: To-Hyun-Sam) has been used as an antipyretic and anti-inflammatory agent in traditional medicine. *S. takesimensis* Nakai (Korean: Seom-Hyun-Sam) is restricted to Ulleung-do Island [16,17]. Although this species is a valuable endemic resource, its medicinal efficacy has not been assessed to date.

The therapeutic potential of *Scrophularia* species is associated with the functions of major secondary metabolites, such as phenylpropanoids and iridoid glycosides, which are present in the plant [18,19]. Harpagoside, an iridoid component present in the *Scrophularia* species, is a bioactive compound of *Harpagophytum procumbens* DC. (Devil’s Claw) and has been used in Southern Africa to treat pain, arthritis, and ulcers. Pharmacological effects of harpagoside on RANKL-induced osteoclast differentiation have also been reported [20,21]. However, there are few reports concerning the pharmacological activity of *Scrophularia* species on RANKL-induced osteoclast differentiation, and studies on the biological activity of *S. koraiensis* and *S. takesimensis* have not been reported.

In the current study, we compared the morphological characteristics and harpagoside content of *S. buergeriana*, *S koraiensis*, and *S. takesimensis*, and compared the effects of *Scrophularia* species extracts on RANKL-mediated osteoclast differentiation.

## 2. Results

### 2.1. Comparative Morphology of Scrophularia Species

**Figure 1:**
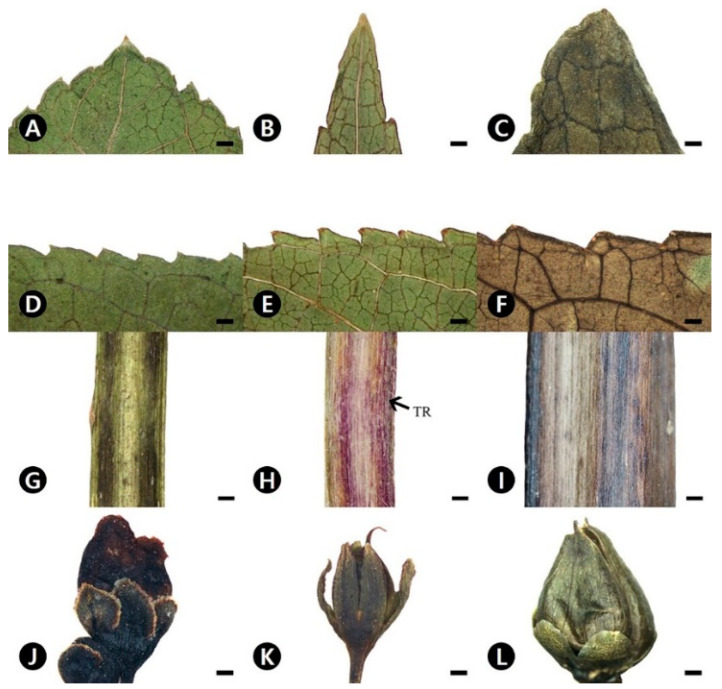
Stereomicroscope micrographs showing the morphology of the three *Scrophularia* species studied. (**A**–**C**) Apex of leaf blade. (**D**–**F**) Margin of leaf blade. (**G**–**I**) Surface of stem. (**J**–**L**) Calyx of flower and/or fruit. (**A**,**D**,**G**,**J**) *S. buergeriana*. (**B**,**E**,**H**,**K**) *S. koraiensis*. (**C**,**F**,**I**,**L**) *S. takesimensis*. TR, Trichomes. All scale bars = 1 mm.

The three species can be distinguished on the basis of leaf shape, apex, margins, pubescence of stems, and calyx shape (Table 1). The leaf blade of *S. buergeriana* is ovate, with an acute apex (Figure 1A), and serrate with a spinose tooth marginal shape (Figure 1D). The stem is glabrous (Figure 1G), and the calyx is ovate with an obtuse apex (Figure 1J). *S. koraiensis* has lanceolate to rarely ovate-shaped leaf blades with an acuminate apex (Figure 1B) and is serrate with a spinose tooth (Figure 1E). The stem of *S. koraiensis* is sparsely pubescent with non-glandular trichomes (Figure 1H), and the calyx is lanceolate with an acute to attenuate apex (Figure 1K). However, *S. takesimensis* has ovate leaf blades with an acute apex (Figure 1C), and has a serrate, almost without spinose tooth, marginal leaf blade (Figure 1F). The stem surface of *S. takesimensis* is glabrous (Figure 1I), and its calyx is semicircular, with a rounded apex (Figure 1L).

### 2.2. Harpagoside Content of Scrophulariae Species

**Figure 2:**
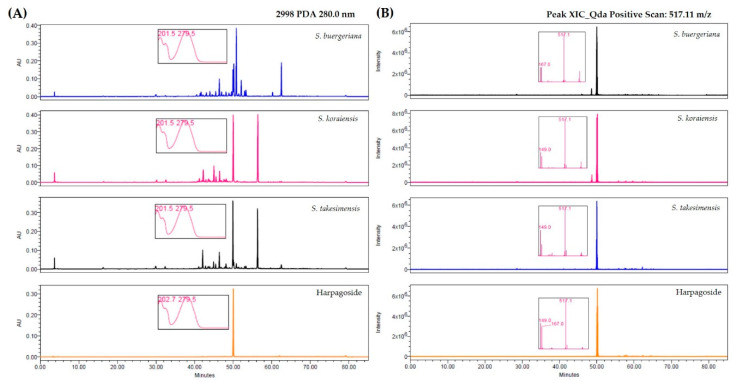
(**A**) Chromatograms of three *Scrophularia* species (*S. buergeriana*, *S. koraiensis*, and *S. takesimensis*) at 280 nm for harpagoside (λ_max_ = 279.5 nm) and (**B**) positive extracted ion chromatogram (XIC) spectrum of harpagoside at 517.11 *m*/*z*.

High-performance liquid chromatography (HPLC) chromatograms of *S. buergeriana*, *S. koraiensis*, and *S. takesimensis* are shown in Figure 2A,B. Figure 2A shows the HPLC chromatograms of the harpagoside standard compound and the three species of *Scrophulariae* monitored at 280 nm, because the maximum wavelength of harpagoside is 279.5 nm. Harpagoside was detected at approximately 50.1 min. The harpagoside content of *S. buergeriana*, *S. koraiensis*, and *S. takesimensis* was 1.94 ± 0.24 mg/g, 6.47 ± 0.02 mg/g, 5.50 ± 0.02 mg/g, respectively (Table 2). The total ion chromatography (TIC) of the mass spectrometry (MS) spectrum was confirmed from 190–850 *m*/*z*, and the extracted ion chromatogram (XIC) of harpagoside in the samples was analyzed at 517.11 *m*/*z*, because harpagoside was detected at 517.11 *m*/*z* [M-H + Na]^+^ (Figure 2B).

### 2.3. Cytotoxic Effects on Primary Murine BMM Growth

**Figure 3:**
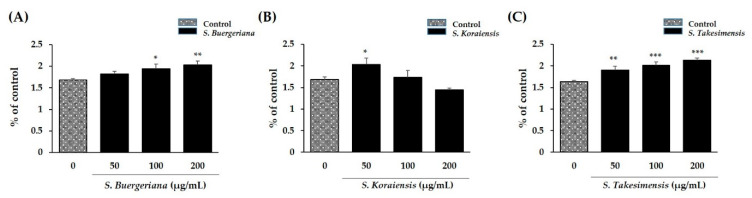
Cell viability affected by ethanol extracts of the three *Scrophularia* species (**A**) *S. buergeriana* (**B**) *S. koraiensis* and (**C**) *S. takesimensis*. * *p* < 0.05, ** *p* < 0.01, and *** *p* < 0.001 vs. control (dimethyl sulfoxide; DMSO).

The XTT assay was conducted to assess cytotoxicity during osteoclast differentiation. *S. buergeriana*, *S. koraiensis*, and *S. takesimensis* did not reduce cell viability at most of the concentrations tested. Treatment with 200 μg/mL *S. koraiensis* reduced cell viability from that of the normal control group (Figure 3A–C), but the difference was not statistically significant.

### 2.4. Effects on Osteoclast Differentiation

**Figure 4:**
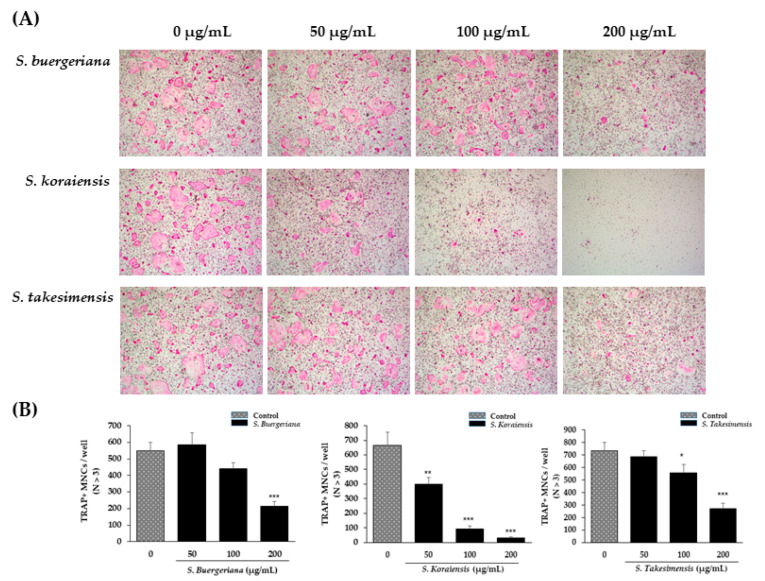
Effects on osteoclast differentiation of ethanol extracts of *Scrophularia buergeriana*, *S. koraiensis*, and *S. takesimensis* at concentrations of 50 μg/mL, 100 μg/mL, and 200 μg/mL. (**A**) Tartrate-resistant acid phosphatase (TRAP)-positive cells photographed (100× magnification) after bone marrow macrophages were cultured with macrophage colony stimulating factor (M-CSF) and nuclear factor (NF)-κB ligand (RANKL) in the presence of ethanol extracts of the three *Scrophularia* species. (**B**) TRAP-positive cells were counted as osteoclasts. * *p* < 0.05, ** *p* < 0.01, and *** *p* < 0.001 vs. control (DMSO).

To compare the effect of *S. buergeriana*, *S. koraiensis*, and *S. takesimensis* on osteoclast differentiation, mouse BMMs treated with macrophage colony stimulating factor (M-CSF) and RANKL were cultured in the presence or absence of ethanol extracts of the three *Scrophularia* species. RANKL and M-CSF induced differentiation of BMMs after incubation for 4 days. TRAP-positive osteoclasts were present in higher numbers in the control group, whereas treatment with *Scrophularia* species extracts inhibited the formation of TRAP-positive cells in a dose-dependent manner (Figure 4B). The *S. koraiensis* treatment group showed suppressed formation of RANKL-induced TRAP activity at concentrations of 100 μg/mL and 200 μg/mL.

### 2.5. Effects on Bone Resorption

**Figure 5:**
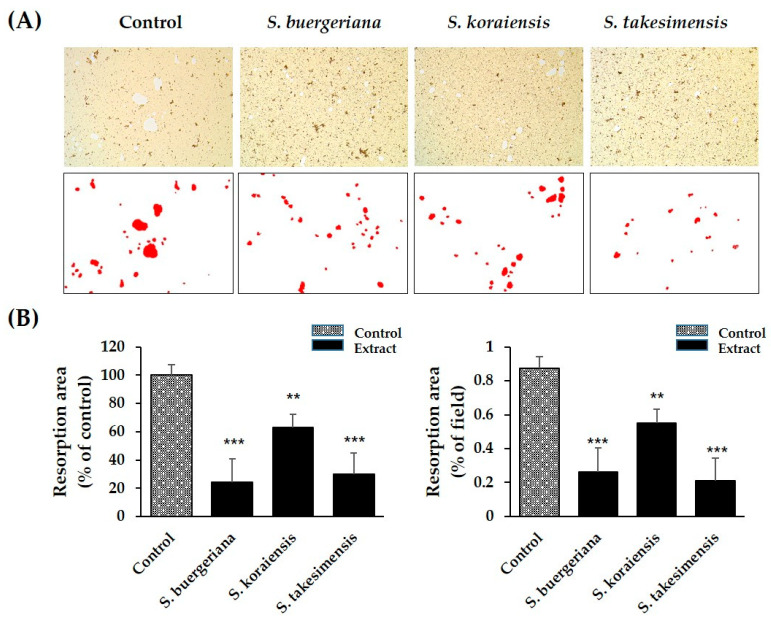
Effects of *S. buergeriana*, *S. koraiensis*, and *S. takesimensis* on bone resorption by mature osteoclasts. (**A**) Hydroxypatite-adherent cells were collected and imaged under a light microscope. (**B**) Resorption areas quantified on hydroxyapatite-coated plates. ** *p* < 0.01 and *** *p* < 0.001 vs. control (DMSO).

To evaluate the effects of *Scrophularia* species on bone resorption, mature osteoclasts and extracts of *S. buergeriana*, *S. koraiensis*, and *S. takesimensis* were applied to plates coated with hydroxyapatite for symbiotic culture of the osteoblasts. Although the area of hydroxyapatite-adsorbed osteoclasts was increased in the control, the resorption area was markedly decreased by treatment with extracts of the three *Scrophularia* species. The resorption inhibition effect was highest for *S. buergeriana*, followed by *S. koraiensis*, and then *S. takesimensis.* (Figure 5A,B).

## 3. Discussion

Osteoporosis, one of the major diseases attracting attention worldwide, is associated with lowered bone mass density as a result of an imbalance between the osteoblasts and osteoclasts that influence bone homeostasis [22,23]. Therapeutic agents such as bisphosphonates are currently used to treat bone resorption diseases, but the long-term use of these drugs leads to the suppression of bone formation and osteonecrosis [24]. Therefore, the beneficial pharmacological effects of plant-derived natural compounds have been advocated [10].

Several studies have reported that iridoid glycosides in *Scrophularia* plant extracts demonstrate anti-inflammatory, neuroinflammation, antioxidant, and hepatoprotective effects [11,12,13,15,25] Harpagoside, an iridoid glycoside, is a main bioactive component of *Harpagophytum procumbens* (family Pedaliaceae) root used to treat chronic rheumatism, osteoarthritis, and arthritis, and is an active constituent of *Scrophularia* species [21,26,27,28]. We quantified the harpagoside content in *S. buergeriana*, *S. koraiensis*, and *S. takesimensis* using HPLC/MS (Table 2, Figure 2). Harpagoside was detected in three *Scrophularia* species, with the highest content found in *S. koraiensis* extract.

In this study, we investigated the inhibitory effects exerted by three *Scrophularia* species containing harpagoside against osteoclast differentiation and bone resorption without cytotoxic effects in RANKL-induced cells. The cytotoxicity of *S. buergeriana*, *S. koraiensis*, and *S. takesimensis* was evaluated in BMMs and measured by XTT assay during osteoclast differentiation. Treatment with ethanol extracts of the three *Scrophularea* species did not reveal cytotoxic effects on BMMs up to 200 μg/mL, with more than 90% cell viability being observed.

RANKL, a bone formation biomarker, is associated with stimulation of osteoblasts and osteoclast differentiation [22]. The balance of bone formation is influenced by osteoblasts and bone resorption activity by osteoclastic cells [29]. Previous studies have demonstrated that harpagoside improved bone properties by inhibiting the formation of osteoclasts from BMMs and the maturation of osteoblast cells [21,30].

TRAP expression is associated with osteoclast maturation and differentiation and is a standard approach to the detection of osteoclasts [24]. In our study, TRAP staining indicated that the numbers and areas of TRAP-positive cells increased, whereas the *S. buergeriana*, *S. koraiensis*, and *S. takesimensis* treatment groups exhibited considerably fewer TRAP-stained osteoclasts, without any cytotoxicity. The results suggested that the three *Scrophularia* species inhibit osteoclast differentiation and formation in BMMs. The inhibitory effect of harpagoside was increased in a dose-dependent manner by downregulating TRAP expression in BMCs [30]. Therefore, it is presumed that the difference in efficacy of the three Scrophularia species in our results would be affected by the content of harpagoside. To study the inhibitory effects of *Scrophularia* species on bone resorption by osteoclast formation induced by RANKL, we investigated bone resorption pit generation in mature osteoclasts. Although the area of hydroxyapatite-absorbed osteoclasts was increased in DMSO controls, the resorption area was markedly decreased by treatment with the three *Scrophularia* species. The highest resorption inhibition effect was exerted by *S. buergeriana*, followed by *S. takesimensis*, and then *S. koraiensis.*

## 4. Materials and Methods

### 4.1. Chemicals

Harpagoside was purchased from Shanghai Sunny Biotech (Shanghai, China). HPLC-grade solvents were obtained from Merck (Darmstadt, Germany). Sodium 3′-[1-(phenyl-aminocarbonyl)-3,4-tetrazolium]-bis(4-methoxy-6-nitro) benzene sulfonic acid hydrate (XTT reagent) was purchased from Sigma-Aldrich (St. Louis, MO, USA).

### 4.2. Plant Materials

Samples of the three *Scrophularia* species *(S. buergeriana*, *S. koraiensis*, and *S. takesimensis*) were obtained from the Department of Herbal Crop Research (Eumseong-gun, Chungcheongbuk-do, Korea). The same vouchers were used for both chemical and morphological analyses (*S. buergeriana*; 2-18-0144, *S. koraiensis*; 2-18-0145, *S. takesimensis*; 2-18-0146) from the Korean Herbarium of Standard Resources (KHSR), Korean Institute of Oriental Medicine.

### 4.3. Comparative Morphology of Scrophularia Species

*S. buergeriana* and *S. koraiensis* were originally collected from the experimental field of the National Institute of Horticultural and Herbal Science (NIHHS, Korea), and *S. takesimensis* was collected from the natural population on Ulleung-do Island. Vouchers of the studied medicinal materials (*S. buergeriana* (2-18-0144), *S. koraiensis* (2-18-0145), and *S. takesimensis* (2-18-016)) were deposited in the Korean Herbarium of Standard Herbal Resources (Index Herbariorum code: KIOM) at the Korea Institute of Oriental Medicine (Naju, Korea). We observed the voucher specimens of the three species (Figure S1) sing an Olympus SZX16 stereomicroscope (Olympus, Tokyo, Japan) for morphological comparison.

### 4.4. HPLC/MS Analysis

*S. buergeriana* (778.14 g), *S. koraiensis* (69.15 g), and *S. takesimensis* (92.5 g) were refluxed in 70% ethanol (*v*/*v*) for 2 h, and these extracts were filtered through filter paper and evaporated in vacuo. The powders of the 70% ethanol extract for *S. buergeriana*, *S. koraiensis*, and *S. takesimensis* were 363.83 g (46.76% of yield, *w*/*w*), 30.40 g (43.96% of yield, *w*/*w*), and 53.91 g (58.28% of yield, *w*/*w*), respectively, and these extracts were stored at 4 °C. Accurately weighed powders of three 70% ethanol extracts (10 mg) were dissolved in 10 mL 70% ethanol and filtered through a 0.2 μm syringe filter prior to HPLC analysis. HPLC was performed using a Waters e2695 Separation Module (Waters Corporation, Milford, MA, USA) and a 2998 photodiode array detector (Waters), combined with an Acquity QDa detector and a micro-splitter (IDEX Health & Science LLC, Oak Harbor, WA, USA) for MS. A Kinetex Phenyl-hexyl 100A column (4.6 × 250 mm, 5 μm, Phenomenex Inc., Torrance, CA, USA) was utilized. The mobile phase was 0.05% aqueous formic acid (A), methanol (B), and acetonitrile (C) using the following gradient program: 100% A (2 min) → 96% A (3% B and 1% C, 7 min) → isocratic 85% A (11% B and 4% C, from 15 min to 20 min) → 70% A (23% B and 7% C, 35 min) → 50% A (35% B and 15% C, 45 min) → 30% A (50% B and 20% C, 55 min). The following conditions were applied: The flow rate was 0.8 mL/min, injected volume was 10 μL, and UV wavelength was monitored from 195 nm to 400 nm. The QDa mass detector employed the following conditions: Nitrogen carrier gas, 0.8 kV electrospray ionization (ESI) capillary, 600 °C probe temperature, 15 V con voltage, 120 °C source temperature, 20:1 split. TIC was monitored from 150 *m*/*z* to 850 *m*/*z*. Harpagoside was detected at 280 nm and 517.11 *m*/*z*.

### 4.5. BMM (Bone Marrow Macrophage) Isolation and Culture

Five-week-old ICR mice were sacrificed by cervical dislocation, the thigh and shin bones were aseptically excised, and the soft tissue was removed. After cutting both ends of the iliac crest, bone marrow cells were obtained by flushing both ends of the bone material using a 1 mL syringe. The isolated BMMs were incubated in a culture dish for 1 day in α-MEM medium containing 10% FMS and 1% penicillin/streptomycin, and unsaturated cells were collected. After 3 days, attached macrophages were used in this experiment. Macrophages were treated with M-CSF (30 ng/mL) and RANKL (100 ng/mL), and incubated with *S. takesimensis*, *S. koraiensis*, and *S. buergeriana* extracts at concentrations of 50 μg/mL, 100 μg/mL, and 200 μg/mL. The day following subculture, the cultured cells were stained with a TRAP solution. Cells with three or more nuclei among red-stained cells were considered differentiated osteoclasts, and the degree of differentiation was measured.

### 4.6. Cell Cytotoxicity

BMMs were seeded in 96-well plates at a density of 1 × 10^4^ cells/well and were treated with M-CSF (30 ng/mL) and 50 μg/mL, 100 μg/mL, and 200 μg/mL ethanol extracts of *Scrophularia* species (*S. takesimensis*, *S. koraiensis*, and *S. buergeriana*) for 3 days. After 3 days, 50 μL XTT reagent (Sigma-Aldrich) was applied for 4 h. The optical density was measured at 450 nm using an ELISA reader (Biotek Instruments, Winooski, VT, USA).

### 4.7. Bone Resorption Pit Assay

To obtain mature osteoclasts, BMMs obtained from the shin and thigh bones of 5-week-old ICR mice and osteoclasts isolated from the skull of 1-day-old ICR mice were added to a 90 mm culture plate coated with collagen. To produce a symbiotic culture, 1α,25-Dihydroxyvitamin D3 (VitD3) and prostaglandin E2 were added. After incubation for 6 days, cells were removed by treatment with 0.1% collagenase and added to a 96-well plate coated with hydroxyapatite. At the same time, *S. takesimensis*, *S. koraiensis*, and *S. buergeriana* extracts were added to the hydroxyapatite-coated plates at a concentration of 200 ug/mL and cultured for 24 h. The cells were washed with distilled water and observed through an optical microscope (Nikon, Tokyo, Japan). The hydroxyapatite-absorbed portion was imaged using micrography, and the area was measured using ImageJ software version 1.51 (National Institutes of Health, Bethesda, MD, USA).

### 4.8. Statistical Analysis

Data were expressed as the mean ± standard deviation (SD). Statistical analysis was performed by analysis of variance (ANOVA), followed by a multiple comparison procedure using Dunnett’s test. A value of *p* < 0.05 indicated significant difference.

## 5. Conclusions

Our results suggest that the *Scrophularia* species may prevent bone loss by inhibiting osteoclast differentiation and born resorption without causing cytotoxicity. It was confirmed that similar metabolites were contained in the *Scrophularia* species, and their extracts exerted similar efficacy. *S. buergeriana* and *S. koraiensis* demonstrated inhibitory effects against osteoclast differentiation and bone resorption and had the highest harpagoside content. Therefore, the three *Scrophularia* species, including *S. koraiensis*, may be potentially therapeutic for treating bone disorder diseases, but the mechanism underlying their bioactivity requires further study.

## Figures and Tables

**Table 1:** Major determinants of *Scrophularia* species.

	Leaves			Stems	Calyx	
	Shape	Apex	Margins	Surfaces	Shape	Apex
*S. buergeriana*	ovate	acute	serrate with spinose tooth	glabrous	ovate	obtuse
*S. koraiensis*	lanceolate to rarely ovate	acuminate	serrate with spinose tooth	pubescent	lanceolate	acute to attenuate
*S. takesimensis*	ovate	acute	serrate almost without spinose tooth	glabrous	semi-circular	rounded

**Table 2:** Harpagoside content of three *Scrophularia* species (*S. buergeriana*, *S. koraiensis*, and *S. takesimensis*) by high-performance liquid chromatography (HPLC) chromatogram analysis.

Scrophulariae Species	Harpagoside (mg/g)
*S. buergeriana*	1.94 ± 0.24 ^(^^1)^
*S. koraiensis*	6.47 ± 0.02
*S. takesimensis*	5.50 ± 0.02

^(1)^ Values are expressed as means ± standard deviation (SD) of three samples for each species.

## Supplementary Materials

The following are available online at https://www.mdpi.com/2223-7747/9/12/1656/s1.

## Abbreviations

BMM, bone marrow-derived macrophage; RANKL, receptor activator of nuclear factor (NF)-κB ligand; TIC, total ion chromatography; TRAP, tartrate-resistant acid phosphatase; XIC, extracted ion chromatogram; XTT, sodium 3′-[1-(phenyl-aminocarbonyl)-3,4-tetrazolium]-bis(4-methoxy-6-nitro) benzene sulfonic acid hydrate.
