# Supplementary material for: Three Scrophularia Species (Scrophularia buergeriana, S. koraiensis, and S. takesimensis) Inhibit RANKL-Induced Osteoclast Differentiation in Bone Marrow-Derived Macrophages
Source: Plants (Basel). 2020 Nov 26;9(12):1656. doi: 10.3390/plants9121656 (PMC7760964; doi:10.3390/plants9121656)
Supplement: Supplementary file 1 [file plants-09-01656-s001.pdf]

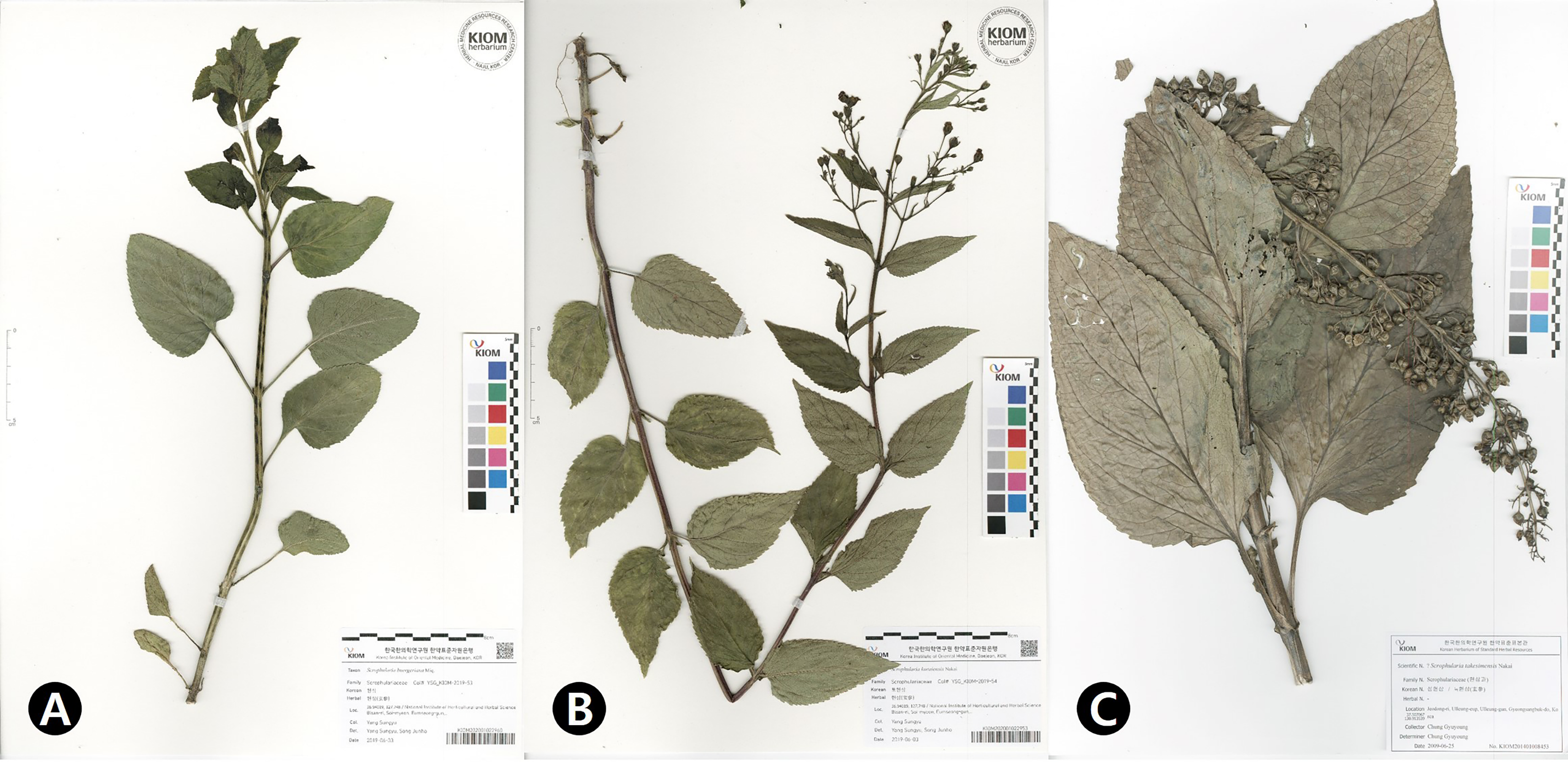
KIOM herbarium
KIOM
A
B
C
8cm
KIOM
한국한의학연구원 한약표준자원은행
Family Scrophulariaceae Col# YSG_KIOM-2019-53
Col. Yang Sungyu
Det. Yang Sungyu, Song Junho
Date 2019-06-03
KIOM202001022960
Scrophularia koraiensis Nakai
Family Scrophulariaceae Col# YSG_KIOM-2019-54
Herbal 현삼(玄參)
Col. Yang Sungyu
Det. Yang Sungyu, Song Junho
Date 2019-06-03
KIOM202001022953
Korea Institute of Oriental Medicine, Daejeon, KOR
Korean Herbarium of Standard Herbal Resources
Scientific N. ? Scrophularia takesimensis Nakai
Family N. Scrophulariaceae (현삼과)
Herbal N. -
Location Jeodong-ri, Ulleung-eup, Ulleung-gun, Gyeongsangbuk-do, Korea
Collector Chung Gyuyoung
Determiner Chung Gyuyoung
Date 2009-06-25
No. KIOM201401008453
